# Pulmonary Alveolar Proteinosis and Pregnancy: A Review of the Literature and Case Presentation

**DOI:** 10.3390/medicina58080984

**Published:** 2022-07-23

**Authors:** Brindusa Ana Cimpoca Raptis, Anca Maria Panaitescu, Gheorghe Peltecu, Nicolae Gica, Radu Botezatu, Mihaela Roxana Popescu, Anca Macri, Ana Constantin, Bogdan Pavel

**Affiliations:** 1 Department of Obstetrics and Gynecology, Carol Davila University of Medicine and Pharmacy, 020021 Bucharest, Romania; brindusa.cimpoca@drd.umfcd.ro (B.A.C.R.); gheorghe.peltecu@umfcd.ro (G.P.); gica.nicolae@umfcd.ro (N.G.); radu.botezatu@umfcd.ro (R.B.); 2 Filantropia Clinical Hospital, 011132 Bucharest, Romania; 3 Cardiothoracic Pathology Department, Carol Davila University of Medicine and Pharmacy, 050474 Bucharest, Romania; roxana.popescu@umfcd.ro; 4 Cardiology Department, Elias Emergency University Hospital, Carol Davila University of Medicine and Pharmacy, 050474 Bucharest, Romania; 5 Department of Pneumology, Marius Nasta National Institute of Pneumology, 050159 Bucharest, Romania; anca.macri@umfcd.ro (A.M.); ana.constantin@umfcd.ro (A.C.); 6 Department of Functional Sciences, Carol Davila University of Medicine and Pharmacy, 050474 Bucharest, Romania; bogdan.pavel@umfcd.ro; 7 Reconstructive Surgery and Burns, Clinical Emergency Hospital of Plastic, 010713 Bucharest, Romania

**Keywords:** pulmonary alveolar proteinosis, pulmonary alveolar lipoproteinosis, pregnancy complications, auto-antibodies, maternal–fetal transfer

## Abstract

Pulmonary Alveolar Proteinosis (PAP) is a rare, usually autoimmune, disease, where surfactant accumulates within alveoli due to decreased clearance, causing dyspnea and hypoxemia. The disease is even more rare in pregnancy; nevertheless, it has been reported in pregnant women and can even appear for the first time during pregnancy as an asthma-like illness. Therefore, awareness is important. Similarly to many autoimmune diseases, it can worsen during pregnancy and postpartum, causing maternal and fetal/neonatal complications. This paper offers a narrative literature review of PAP and pregnancy, while illustrating a case of a pregnant patient with known PAP who developed preeclampsia in the third trimester but had an overall fortunate maternal and neonatal outcome.

## 1. Introduction

Pulmonary Alveolar Proteinosis (PAP) is a rare disease also known as pulmonary alveolar lipoproteinosis or phospholipidosis. Surfactant accumulates within alveoli due to decreased clearance rather than increased production [1]. This surfactant blocks air from entering alveoli and oxygen from passing through into the blood, which results in dyspnea and hypoxemia. This condition is subdivided into primary (autoimmune), secondary (in the context of other systemic diseases), or congenital (usually genetic), depending on the pathophysiologic mechanism.

**Figure 1:**
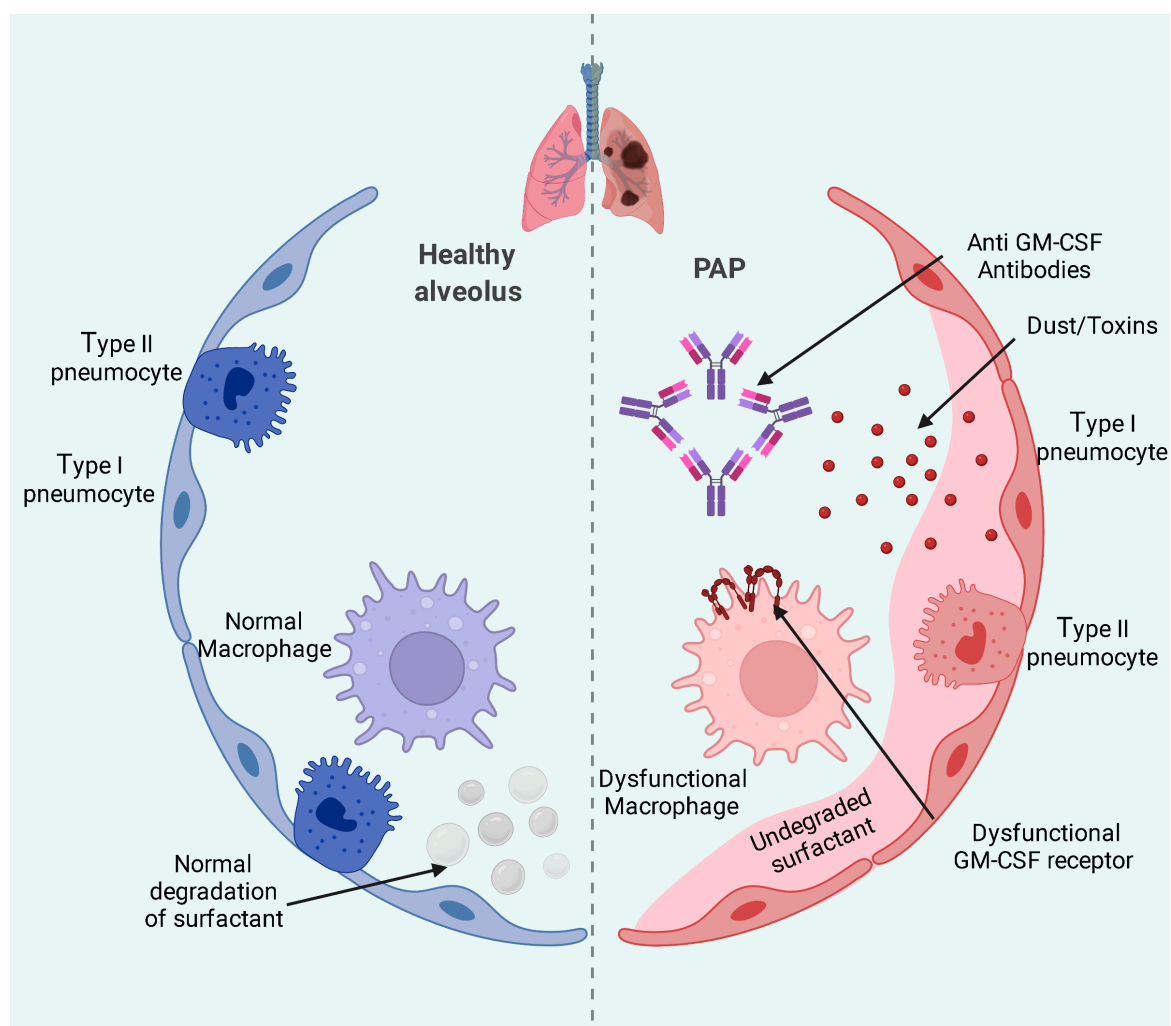
Normal surfactant clearance (blue) and changes occurring in PAP (red). In PAP, there are abnormal antibodies against the granulocyte macrophage colony stimulating factor that impair the normal function of lung macrophages with the accumulation of undegraded surfactant. Gas exchange is altered at the level of the lung alveoli, and this can lead to hypoxemia.

Autoimmune PAP is the most common form, accounting for 90% of all known cases. It is mediated by immunoglobulin G (IgG) anti-granulocyte macrophage colony stimulating factor (anti-GM-CSF) antibodies, which cause a decrease in alveolar macrophages’ function [2]. Increased levels of anti-GM-CSF are found in the bronchoalveolar lavage fluid in PAP patients [1], and the diagnosis is based on the examination of this fluid or transbronchial biopsies [2]. Surfactant clearance under normal conditions and abnormal clearance in PAP is illustrated in Figure 1.

In secondary PAP, the macrophages have decreased functions caused by hematological malignancies, such as myelodysplastic syndrome or chronic myelogenous leukemia, and not by anti-GM-CSF antibodies. Primary immunodeficiency diseases or genetic syndromes, such as DiGeorge syndrome, can also cause secondary PAP [3,4].

Congenital PAP is the least common form and is caused by a genetic mutation in GM-CSF receptor proteins or surfactant proteins [5]. It is inherited in either an autosomal dominant (AD), autosomal recessive (AR), or X-linked recessive order depending on the faulty gene [6]. The human receptor for granulocyte macrophage colony stimulating factor is composed of an alpha, which corresponds to CSF2RA, and a beta subunit, which corresponds to CSF2RB [5]. PAP can be caused also by mutations in surfactant protein B (SFTP3-gene), surfactant protein C, ATP-binding cassette 3, or NK2 homeobox, resulting in a dysfunctional surfactant release from type II epithelial cells and abnormal clearance from the alveoli, leading to the accumulation of lipoproteinaceous deposits in the alveoli [7,8]. The above-mentioned causes of PAP are summarized in Table 1.

The evolution of PAP is variable and unpredictable, ranging from spontaneous remission to progression towards chronic severe respiratory failure [9].

The gold standard treatment is symptomatic whole-lung lavage (WLL) [10]. Many therapies aiming to improve surfactant clearance have been investigated, either by targeting alveolar macrophages with exogenous GM-CSF or aiming to reduce anti-GM-CSF antibodies levels with plasmapheresis or rituximab. Nevertheless, the exact location of these treatments is not currently well defined [3].

Pulmonary alveolar proteinosis is even more rare in pregnancy, and obstetricians are not familiar with this disease; nevertheless, the condition has been reported in pregnant women and can even appear for the first-time during pregnancy, usually as an asthma-like illness; [11] therefore, awareness is important.

The aim of this paper is to discuss the implications of PAP in pregnancy, and to present a case of a pregnant woman with PAP that had a fortunate outcome.

## 2. Material and Methods

Data collection was performed using international databases PubMed, Scopus, and Web of Science until May 2022, searching for reports dealing with PAP and pregnancy using the following key words: pulmonary alveolar proteinosis and pregnancy.

## 3. Results

Only a handful of cases of PAP during pregnancy has been reported so far in the literature, accounting for only 12 entries in Web of Science and 21 in PubMed. After reviewing these papers, we identified four documents reporting cases of PAP in pregnancy with maternal and neonatal outcomes.

Cases published so far are summarized in Table 2.

In three cases, WLL was required during pregnancy, while in one case with moderate to restrictive ventilatory defect, there were no exacerbations of PAP. Vaginal delivery was the method of birth in two cases, including a pair of twins, while in the more recently reported cases, Caesarean section was preferred. In three of the cases, pregnancies advanced to full term.

## 4. Discussion

Pulmonary alveolar proteinosis is a rare condition mostly in the context of autoimmunity against the anti-granulocyte macrophage colony-stimulating factor. It is even more rarely encountered in pregnancy. As many autoimmune diseases, PAP can worsen during pregnancy or postpartum, causing maternal and fetal complications. Furthermore, the abnormal autoantibodies of the IgG class, such as those seen with PAP, can cross the placenta and theoretically can harm the fetal lung development, as it does happen in other maternal autoimmune conditions [16,17,18].

### 4.1. Effect of Pregnancy on PAP

During pregnancy, there are several physiologic adaptative changes of the respiratory function that occur: minute ventilation increases, respiratory rate increases, functional residual capacity (FRC) is reduced, and oxygen consumption increases [19]. These changes are summarized in Table 3.

During pregnancy, PAP symptoms can be exacerbated, and WLL may be necessary to improve maternal hypoxia. The indication of therapy must be considered by a multidisciplinary team including internal medicine specialists, anesthesiologists, obstetricians, fetal medicine specialists, and neonatologists. So far in the literature, there are few cases of WLL in pregnancy described, and mainly before 34 weeks gestation, as shown in Table 3 [12,13,14,15]. Among those cases, all but one experienced the exacerbation of the disease during pregnancy [14]. The most extensive survey on PAP patients treated with WLL, conducted by Campo et al., showed that most common complications related to this procedure are, in order of appearance, the following: fever (less than 20%), hypoxemia, pneumonia (about 5%), pleural effusion, and, rarely, pneumothorax [10]. With advanced gestation and the increasing uterine volume, WLL is more challenging, and the risks and benefits should be discussed with the patient and an informed decision must be made regarding the treatment options. If delivery is required before 34 weeks, maternal steroids for fetal lung maturation should be offered to every patient with PAP [20].

PAP during pregnancy can be complicated by opportunistic infections due to the reduced rate of clearance. Macrophages are unable to clear the surfactant and are less efficient against infections [21]. About 10% of patients with autoimmune PAP develop secondary infections [22], of which more than two-thirds are tuberculosis [23]. It is expected that during gestation, medical treatment or prophylaxis for most opportunistic infections is similar to that for non-pregnant adults. In pregnancy, the infection rate may be higher. The possible explanation relies on pregnancy-related intrinsic immunosuppression, especially T-cell dysfunction among decreased alveoli clearance [24,25].

### 4.2. Effect of PAP on Pregnancy

Autoimmune PAP can have consequences on pregnancy due to maternal pulmonary insufficiency and hypoxemia. Fetal growth restriction and maternal preeclampsia are among the most common pregnancy complications in mothers with PAP. Human neonates demonstrate low birth weight or growth restriction due to their antenatal hypoxic environment [26]. Maternal low oxygen levels alter fetal cardiac growth and neonatal vascular function, causing fetal growth restriction, and in more severe cases, permanent neurological deficit or pulmonary dysfunction [27]. Autoimmune diseases might harbor an increased susceptibility to preeclampsia. Among the immunological abnormalities, aberrant natural killer cell biology has also been implicated in the endothelial dysfunction related to preeclampsia [28]. So far, there are no specific studies demonstrating the link between autoimmune PAP and preeclampsia; however, growing evidence reveals the important role of maternal hypoxia in preeclampsia [29,30]. Preeclampsia is associated with inadequate trophoblast invasion and poor spiral arteries remodeling leading to placental hypoxia. However, whether maternal hypoxia in patients with PAP is a main agent in the pathogenesis of preeclampsia remains unclear, as not all PAP patients develop preeclampsia. The possible explanation might be the difference in the abilities of the compensatory response to chronic hypoxia among different women with PAP [31]. Moreover, autoimmune disorders increase the risk of general autoimmunity, and patients with PAP should be evaluated for co-existence of other immune abnormalities that can interfere with pregnancy.

Pulmonary alveolar proteinosis can appear for the first time in pregnancy and can easily be mistaken for other more prevalent pulmonary diseases such as asthma, pulmonary hypertension, or pulmonary embolism, and more recently, COVID-19 [32,33,34,35]. Due to the limited possibility of the use of all diagnostic techniques during pregnancy, the correct diagnostic could be delayed.

Moreover, a worthy discussion involves genetic advice in families where PAP is documented, as reports show that homozygous newborns from heterozygous parents have a negative prognosis [36]. Congenital PAP is an inherited disease where the affected fetus unfortunately dies within the first months of life due to deficiency of surfactant protein B (SP-B), causing imbalanced surfactant homeostasis and function, which leads to respiratory failure [37]. Stuhrmann reported a case where both parents were heterozygous for the SFTP3-gene, and unfortunately, the fetus was homozygous for the faulty gene on prenatal chorionic villus sampling [37].

### 4.3. Case Report

We present the case of a 31-year-old, para 1, gravida 1 woman with pulmonary alveolar proteinosis who opted for pregnancy surveillance in our unit. Autoimmune PAP was diagnosed during late adolescence (2006), and the patient required serial whole-lung lavages (WLL) aiming to improve respiratory function, offering a normal life with moderate physical activity. Her last lavage was performed in 2019. She is the only family member affected by PAP, and she is a non-smoker and has a normal body mass index. In 2008, she suffered a cerebral abscess, which was drained and healed without consequences.

The pregnancy resulted after spontaneous conception. First trimester screening showed low risk for aneuploidies, and the fetal anatomy check was unremarkable; only fetal single umbilical artery (SUA) was noted. Maternal screening for preeclampsia showed high risk for preeclampsia before 34 weeks. Blood pressure was within normal limits. Prophylactic aspirin 150 mg/day commenced at 12 weeks of pregnancy. The mother was counselled regarding pregnancy complications and the risk of worsening of pulmonary function during pregnancy, reaching a peak after 34 weeks of gestation.

The mid-pregnancy anatomy check was within normal limits; fetal growth was plotted around the 10th centile in the third trimester. Maternal steroids for lung maturation were offered and administrated at 32 weeks, after fetal biometry plotted on the 8th centile and Doppler studies showed increased pulsatility index (PI) in the single umbilical artery.

At 33 weeks and 6 days, blood pressure reached 160/100 mmHg and the mother became oxygen-dependent after minimal physical activity, reaching an oxygen saturation (SpO_2_) of 98–99 under 3–3.5 L/min oxygen/hour. Upon admission, fetal biometry was plotted on the 6th centile and the umbilical artery PI was above the 90th centile, with positive-end diastolic flow. An uneventful C-section was performed and a living 2000 g male baby was born, who was discharged home on day 14. The mother remained under low oxygen requirement (2.5–3 L/min) to reach normal oxygen saturation after moderate physical activity. Blood pressure normalized, and no medication was necessary after discharge.

The 6-week postpartum visit was unremarkable, and the mother was oxygen-dependent only after vigorous physical activity.

### 4.4. Integrative Discussion

A pregnant woman with PAP is prone to hypoxemia by three mechanisms: low functional residual capacity (FRC) due to physiological changes in pregnancy, an increased resistance to diffusion (due to proteinaceous material within alveoli), which associates an increased alveolar-arterial difference of oxygen, and in later stages, the intrapulmonary shunt (when the alveoli are filled with proteinaceous material and cannot be ventilated) [38,39,40]. Since it is known that oxygen consumption (VO_2_) is high in pregnancy, a pregnant woman presenting PAP will desaturate very quickly. Preoperative non-invasive investigations include the following: blood respiratory gases analysis (PaO_2_ should be >65 mmHg), spirometry (in order to assess the vital capacity), and the diffusing capacity for carbon monoxide-DLCO [41]. In a case of anesthesia for Caesarean sections, a hypoxemic event could occur, especially in a case of general anesthesia, and it can also occur during regional anesthesia. In order to prevent a hypoxemic event, especially for the infant, two measures are recommended: preoxygenation with pure oxygen for at least 3 min in order to produce the denitrogenation of the lungs and filling with pure oxygen and continuous positive airway pressure-CPAP sessions before scheduled intervention, knowing that CPAP has the role to increase FRC by recruiting some alveoli; pregnant women are prone to atelectasis [42]. Preoperatively, CPAP is proven to improve the outcome in patients with COPD by increasing FRC and preventing rapid falls in arterial oxygen saturation [42]. Another key point is the quick extraction of the baby by the obstetrician in order to avoid a possible exposure to maternal hypoxemia. A CPAP session could be also recommended before and after bronchoalveolar lavage.

Regarding other therapeutic options, plasmapheresis has a recognized role in autoimmune diseases; thus, theoretically, this therapy could potentially have a good impact on PAP. According to the literature, there are few data about the usage of this therapy in PAP, and there is no consensus regarding clinical improvement [43]. In one case report, clinical improvement was observed after a combined therapy of plasmapheresis and rituximab [44]. Although it seems that plasmapheresis is safe in pregnancy, there is no report about plasmapheresis use in cases of PAP until now [45]. One the other hand, rituximab does not seem as safe as plasmapheresis in pregnancy, as some structural changes in newborns have been reported to be linked to its use [46]. Inhaled GM-CSF was used in cases of PAP with some improvements in lung function [47].

We acknowledge that, in the presented case, the pregnancy and maternal PAP were uneventful and without important maternal clinical manifestations of hypoxia, although the last WLL was performed 3 years prior to pregnancy. Although aspirin prophylaxis was initiated in the first trimester [48], the patient developed PE, raising the question of a possible alternative pathophysiology of preeclampsia in PAP that could be related to immunological abnormalities rather than defective placentation.

## 5. Conclusions

A pregnancy complicated with PAP is a very rare case, and a multidisciplinary medical team including an obstetrician, an anesthesiologist, and a pneumologist should be convened in order to establish a medical plan of investigations and interventions to prevent the occurrence of a hypoxemic event. Worsening maternal conditions can happen at any time but occur more frequently in the third trimester due to the changes in lung function related to the growing uterus. Preeclampsia and fetal growth restriction can occur in these cases possibly due to the hypoxemic environment, and aspirin prophylaxis may also be taken into consideration. In recent literature reports, Caesarean birth has been the preferred option for delivery. International and national registries of cases of PAP in pregnancy will further increase knowledge and awareness on this rare condition.

## Figures and Tables

**Table 1:** Causes of PAP.

Primary	Secondary	Genetic
IgG anti GM-CSF	Kaolin, indium, titanium, talc, silica, aluminum	SFTPB
Deficiency in GM-CSF receptors	HIV infection	SFTPC
	Chemotherapy	NKX2-1
	Organ transplantation	ABCA3
	Agammaglobulinemia	SLC7A7
	Dermatomyositis	CSF2RA
	Rheumatoid arthritis	
	Behcet’s disease	

SFTB—Surfactant protein B; SFTC—Surfactant protein C; NKX2-1—NK2 homeobox 1; ABCA-3—ATP binding cassette 3; SLC7A7—Solute Carrier Family 7 Member 7; CSF2RA—Colony Stimulating Factor 2 Receptor Subunit Alpha.

**Table 2:** PAP and pregnancy literature cases.

Author	Year	Maternal Outcome	Fetal Outcome
Matuschak GM [12]	1984	Twice WLL during pregnancy	Vaginal birth DCDA twins 38 weeks 2097 g and 1360 g
Canto MJ [13]	1995	Moderate restrictive ventilatory defect, without exacerbation during pregnancy	Vaginal birth, Liveborn 1800 g Apgar 8/10
Jannkowich M [14]	2006	Second trimester: segmental lung lavage via flexible bronchoscopy	CS 32 weeks, healthy baby
Belchior I [15]	2011	First trimester: one WLL	CS 37 weeks, healthy baby

WLL—whole-lung lavage; DCDA—dichorionic diamniotic; CS—Cesarean section.

**Table 3:** Respiratory changes during pregnancy.

Respiratory Parameter	Change
Tidal Volume	+40%
Minute Ventilation	+50%
Respiratory rate	+15%
Functional residual capacity	−20%
VO_2_	+20–25%
P_50_	+3 mmHg
FEV1	No change

VO_2_—oxygen consumption; P_50_—paO_2_ corresponding to a saturation of 50%; FEV1—forced expiratory volume at 1 s of expiration.

## Data Availability

Not applicable.
